# Puerperal Peritonitis

**Published:** 1856-06

**Authors:** 


### SIMPLE AND PUERPERAL PERITONITIS.

Transactions of the College of Physicians, Philadelphia.

Dr. Keating inquired whether cases of simple idiopathic peritonitis, or of the puerperal form had fallen under the observation of the Fellows? He had, himself, met with one of the former description.

Dr. Wallace:—About three weeks ago I was called to see a young man at a boarding house. It was on Friday. On the previous Tuesday evening he was well. I am unable to state from observation what his symptoms were in the interval, but when I saw him his pulse was thready, and his abdomen distended, but free from any spontaneous pain. He had been sitting up by the stove because he felt chilly. On the same evening he died.

Examination *post-mortem* revealed the existence of intense and general peritonitis. Indeed, the whole of the parietal as well as the visceral layer was inflamed, the subserous cellular tissue injected, and the cavity of the membrane was filled with a sero-purulent liquid. The rapidity of the course taken by the disease had led me to suspect that it might possibly be due to some traumatic cause, but no perforation of the bowel or other similar lesion could be detected. I therefore regarded the case as one of idiopsthic peritonitis.

In regard to the puerperal form of peritoneal inflammation, I may remark that I have met with two cases, and, as in one of them the course of the disease was somewhat unusual, and as I was able to obtain a *post-mortem* inspection of the body, a brief account of it may not be uninteresting.

On a Sunday morning the woman was confined, and her labor was quite regular in all respects. On the afternoon of Tuesday she had a slight chill and complained of pain in the right groin, which extended upwards, but it soon afterwards subsided. The secretion of milk was abundant, the skin moist, and the lochia but slightly diminished. In fact there was no symptom besides the pain alluded to. Pressure on the abdomen was not very painful. On Wednesday morning the breasts were full, and the general condition excellent; but the lochia were diminished, and the pain in the groin was more readily developed by pressure. At the fundus of the uterus, also, there was abnormal tenderness. The pulse was soft, perhaps twenty beats more frequent than before, but not excited. Leeches were ordered to be applied, and calomel with Dover's powder was given internally. Warm terebinthinate stupes were also used as an application to the abdomen. A few hours afterwards the pain had disappeared, but a state of collapse had eusued with sunken countenance, frequent pulse, etc. In the evening the pulse rallied and beat 120 a minnte. The pain, however, returned, and a renewed application of leeches was thought advisable.

On Thurseay evening the pulse reached 170, but it afterwards declined, and the patient died on Friday night.

I was struck by the extreme mildness, in this case, of all the symptoms which are most characteristic of puerperal peritonitis, and believed that its termination would be favorable. But from the time when the leeches were applied, the countenance changed, and all the evidences of a mortal issue developed themselves.

An examination was made after death. The right ovary was covered with lymph, and filled with small and separate collections of pus; its tissue was readily torn, it was even less firm than a clot of blood found in the cavity of the uterus. This latter had no offensive smell. The left ovary was highly congested, and the corresponding Fallopian tube was of a chocolate color. There was no disordered condition of the uterus, and only *slight* peritoneal inflammation of its serous investment. But all the rest of the peritoneal membrane was intensely inflamed, and there was a large amount of serum, lymph, and pus in the cavity.

An interesting question presents itself: when did the ovarian inflammation begin? Did a condition exist previous to labor which tended to produce the subsequent disorganization? I am rather disposed to believe that it dated from the time of the chill, and I also suspect that the leeching, performed when it was, exerted an injurious influence. I am also inclined to think that in a similar case, it would be my duty to bleed with the first appearance of the local symptoms, with the view of checking or preventing a complete peritoneal inflammation, which, in this case, caused death, and which I believe did insidiously commence when the local symptoms first appeared.

Dr. Keating:—In Dr. Wallace's case I cannot believe that such an amount of local inflammation and of such a grade, could have sprung up so suddenly, without producing more local and constitutional disturbance. I cannot account for such results as were exhibited in his case, except by supposing that a diseased condition existed previous to delivery, or that a latent inflammation had previously existed, and that the chill, instead of announcing the onset of the disease, merely indicated that it had already progressed to the formation of pus. I am confirmed in this belief by the fact that I have notes of three cases in which the patients had, during the last weeks of gestation, complained of sharp lancinating pains over that region, or that ovary, which was the identical spot in which the disease manifested itself after delivery.

It seems to me that during an epidemic of this fatal malady it would be important for medical men to examine carefully the internal organs of generation of all puerperal women who might die during gestation; careful observations, under these circumstances, might prove whether or not this fearful poison had stealthily crept into the patient's blood before her delivery, and was only biding its time to destroy its victim.

So far from my experience confirming the propriety of copious venesection in these cases, I am more and more persuaded that those benefitted by it are frank phlegmasiæ, the most amenable to treatment generally, and differing in every character from that fearful blood-poisoning which prevails epidemically, and defies all treatment.

I cannot agree with the views just offered by Dr. Wallace in reference to bleeding in these cases.

In a recent case I was called in half an hour after the occurrence of the chill. The patient had been delivered forty-eight hours. I resorted to copious venesections, say f℥xx which immediately relieved the pain in the left iliac region, and reduced the pulse from 120 to 110 beats. The blood was neither cupped nor buffed. Immediately after bleeding, I gave a combination of opium and calomel, and applied warm fomentations to the abdomen. Prof. Hodge saw the patient, in consultation with me, three hours after; her pulse had already commenced to flag, and in six hours after, we had to resort to stimulants. I had never previously seen a case so soon after the attack, and never depleted so freely, and I can conscientiously affirm that I never saw a case sink so rapidly.

Dr. Coates:—Gentlemen should not blame themselves for the untoward event, in such a case as that related by Dr. Wallace, any more than in that of sloughing of the intestine, previously described by Dr. Gobrecht; nor does it follow that a contrary practice would have been more successful. The opposite of wrong, in matters of conduct, is not right, but another kind of wrong. It is notoriously beyond our power to prevent a fatal result in many such cases. I do not believe that it is possible, by bleeding largely, to insure or even afford any strong presumption of the cutting short of this disease.

I do not see the evidence that the occurrence of chills is necessarily indicative of the formation of pus in the case. Further, I am unable to discern the grounds in which it is supposed that the ovarian inflammation may not have existed previously to labor. The ovarium is an irritable part of the body, and it is very common for it to undergo great anatomical changes without the production of fever; even peritonitis does not always produce pain or fever. The cases, I believe, are easy of access; those which occur to my memory are from *Broussais's Phlegmasies Chroniques.*

Peritonitis may be induced by cold. In an instance under my own care, a case of rapid and mortal peritonitis was induced in a boy, by his being employed to clean out a bath-tub, in cold weather. Being fond of the cold bath, he remained, a long time amusing himself amid the water. The marks of severe inflammation, in this case, lined the whole abdominal cavity and viscera, extending over the whole peritoneal surface of both sides of the liver—an appearance which I do not recollect having witnessed in any other instance. Where is the evidence that incidental and even apparently slight causes, an exposure to cold air and damp, for example, may not produce peritonitis after labor, when the susceptibility of the organs concerned in that function is, of course, extreme?

Dr. Beesley:—The case just related by Dr. Wallace is of a deeply interesting character—such a serious result following on symptoms apparently so little alarming. The difficulty is great of distinguishing such cases from those attended with similar symptoms, yet arising rather from irritation than inflammation. I may, in elucidation, mention a case which I recently attended. A young and delicate married lady was delivered of her first-born, after eight months' pregnancy. She went on favorably until the fifth day, when she was seized with a chill, headache, and pain in the left iliac region. I saw her about two hours afterward. She was lying on her back with her knees drawn up; complained of considerable pain and soreness on pressure. Abdomen somewhat tumid; had no stool since delivery; her nurse having been too ill to give her the needful attention. Her pulse was 110 and small, rising to 120 in the course of a few hours. Lochia free and milk abundant. She had daily applied cleansing ablutions externally, but a putrid odor was evident.

I directed at once a purgative of calomel, jalap, and aloes, to be followed in four hours, if no stool, by an enema.

The neutral mixture with elixir of opium was also given about every two hours, and warm stupes over the abdomen. Considerable relief was experienced in her feelings after the second stool, but the abdominal tenderness though lessened, continued until the third day, and the pulse was above 100 for two or three days longer. On the second day Dover's powders, in four grain doses every four hours, were substituted for the elixir of opium. Perspiration came on gently and was kept up by this treatment, and my patient was soon convalescent.

In the early years of my practice, I adopted the plan recommended by Dr. Dewees, and bled and purged freely in such cases, and I regret to say, with not that success I desired. But for the last eight years I treat them differently, seldom taking any blood from them, unless the pulse is full and strong, and then rarely more than 16 to 20 oz.

I usually commence with from 12 to 15 grs. calomel, with two grs. of opium, and follow up with four grs. of Dover's powders, every four hours, neutral mixture two hours, and flannels wrung out of warm water to the abdomen, covered with silk oil-cloth and dry flannel. I occasionally use some laxative or an enema to aid the calomel, but generally find that two or three stools of a dark color appear without their assistance, in twenty-four hours. All of these cases which I have seen from the commencement have recovered. Where I suspect putrid blood remains in the uterus, I occasionally use, I think with great advantage, injections of warm water into its cavity, by means of a metallic pipe introduced along the finger into the os uteri, from a glass syringe.

I fear that a poison introduced into the circulation from putrid matters may induce or aggravate the symptoms, and where these appear threatening a few days after delivery, I have seen no evil, but, on the contrary, favorable results from the practice.

Dr. Griscom:—I do not feel quite satisfied that the use of the syringe which has been described is altogether free from danger. In some *post-mortem* examinations of the recently delivered uterus, I have found the Fallopian tubes large enough to admit the little finger, and therefore consider that the operation referred to might cause liquid to be thrown into the peritoneal cavity.

In reference to large bleedings in the disease under consideration, my experience is adverse to their employment. In two cases which occur to my mind, the attending physician bled so as to induce a completely anemic state; but both patients died.

Dr. Beesley:—The degree of openness of the Fallopian tubes after death, is no evidence, to my mind, that they are open during life, and soon after delivery. Were they in that condition, we should have two streams thrown into the cavity of the abdomen, when the uterus acted on fluid blood, at the time that its os is closed, or filled up with a coagulum.

Dr. Condie:—During a practice of thirty-nine years, I have seen enough of puerperal fever to strengthen my adherence to the belief, confirmed now by the conclusions of obstetricians in every part of Europe, and by the majority of those in our own country, that bleeding in this disease is altogether mischievous. It should never be forgotten that when the disease prevails, epidemically, the majority of patients will in general die, whatever may be the plan of treatment pursued.

If the views entertained by our European brethren are correct, puerperal fever is not a local disease, but a blood disease. Acute peritonitis may occur in the puerperal state and be cured by bleeding, but of genuine puerperal fever I defy any one to cure one case out of ten by antiphlogistic remedies. The only treatment of this disease, which can boast of any degree of success, is that which consists mainly in the use of the muriated tincture of iron. It has unfortunately happened that diseases of very dissimilar character and demanding very different plans of treatment, have all been classed under the one general denomination of puerperal fever, merely from the fact of their occurring soon after parturition.

In regard to the suggestion that clots retained in the womb and there becoming putrid may occasion the disease in question, I would remark that in this manner typhus, but not true puerperal fever, may be generated. Nor do I believe that the removal of retained clots by injections thrown into the cavity of the uterus would prevent the development, or retard the fatal issue of genuine puerperal fever for a single hour. Besides, the danger of throwing an injection into the peritoneal cavity is a very serious one, for experiments made in France have shown that such injections are liable to pass from the uterus through the Fallopian tubes into the peritoneal cavity.

As regards the mild treatment recommended under the circumstances pointed out by Dr. Beesley, I fully admit its propriety. Indeed, it was but yesterday that a case occurred to me, presenting the symptoms alluded to, and I obtained a similar result, by adopting the same simple plan of medication; cases such as these, however, cannot be considered as even mild attacks of puerperal fever.

The question at present of interest to us is, does idiopathic erysipelas now prevail in any part of the city simultaneously, with puerperal fever? I have seen several cases of the former disease myself, in the lower part of the city, and have been informed that both it and idiopathic peritonitis have been observed by other physicians in the same neighborhoods.

Dr. Henry Hartshorne mentioned that he was attending, at the present time, a woman in the southwestern part of the city, recovering from symptoms ordinarily considered those of puerperal fever, after having been *bled twice*. He alluded to it because a majority of the testimony, this evening, had been against bleeding in puerperal cases. The amount of blood taken in this case was moderate, ℥xij the first, and ℥xvj the second time; and Dr. H. believed that the treatment which affords the best hope of cure in the greatest number of cases, is what we may designate in one word, as the moderate treatment: in proportion to the symptoms of the case. Bleeding, calomel, and opium, purgation and blistering, all aided in the treatment of the patient alluded to, who was affected with what, from Hospital experience, Dr. H. should have considered very serious symptoms; but none of these remedies were carried to an *extreme* point. While recognizing, with Dr. Condie, the existence of several pathological states under what we commonly call "puerperal fever," *e. g.* simple peritonitis, phlebitis, pyæmia, and epidemic or endemic puerperal fever, with or without peritonitis as a symptom, Dr. Hartshorne could not yet surmount the difficulty of distinguishing these in all cases, and especially at the time when it is most important, during the first day of the disease. He believed that we rarely have cases in which one of these conditions (unless it be simple peritonitis,) is isolated altogether from the others, so as to present unmistakable signs at an early stage. We see a resemblance in this, to erysipelas, in which the constitutional and local elements of the disease unite; sometimes the eruption, and sometimes the general disorder being prominent, and with this difference the treatment must vary essentially.

